# Source-Level Resting-State EEG Connectivity Reveals Frequency-Specific Neural Reorganization and Predicts Motor Recovery in Individuals Post Stroke Following Gait Rehabilitation

**DOI:** 10.21203/rs.3.rs-7411628/v1

**Published:** 2025-12-09

**Authors:** Fares Al-Shargie, Michael Glassen, Gregory R. Ames, Christina M. Dandola, Karen J. Nolan, Soha Saleh

**Affiliations:** Rutgers University; Rutgers University; Kessler Foundation; Kessler Foundation; Kessler Foundation; Rutgers University

**Keywords:** Stroke, Rehabilitation, Exoskeleton, Gait, EEG, Rest State Connectivity, Asymmetry, Machine Learning

## Abstract

**Introduction:**

Stroke-induced motor impairments are linked to disrupted cortical connectivity and remain a major challenge in neurorehabilitation. The purpose of this investigation was to evaluate brain network reorganization and its relationship to motor recovery following a 10-week gait rehabilitation program (3 days/week for a total of 30 sessions).

**Methods:**

Forty-four participants (22 healthy adults HC, 22 individuals diagnosed with stroke) were enrolled, individuals in the stroke group were randomized into Exoskeleton-Assisted Rehabilitation (ER=9) or Standard of Care (SOC=13) groups. Resting-state EEG was recorded pre- and post-intervention. Directed functional connectivity was estimated from localized sources using Partial Directed Coherence (PDC), followed by graph theory analysis, laterality index (LI) computation, and machine learning classification using frequency-specific EEG features.

**Results:**

At baseline, individuals in the stroke group demonstrated reduced connectivity relative to HC group in the affected supplementary motor area (SMA, p<0.005) and unaffected ventral premotor cortex (vPM, p<0.01), with compensatory increases in the affected insula (INS) and dorsalA6. Post-intervention, connectivity increased from the unaffected primary motor cortex (M1) and affected postcentral gyrus (PoG, p<0.01), with dorsalA6 showing enhanced node strength (p<0.01). LI analysis indicated reduced contralesional compensation in cingulate gyrus (CG) and middle temporal gyrus (MTG), and re-engagement of the affected hemisphere (PoG, dorsalA6). DorsalA6 connectivity correlated with Fugl-Meyer score improvements (r=0.64, p=0.001). Machine learning achieved 93.18% accuracy and 94.83% Area Under ROC Curve to differentiate between the groups with features from the alpha and gamma being the most discriminative.

**Conclusions:**

Source-level EEG connectivity and frequency-specific features are sensitive biomarkers of neuroplasticity. The 10-week program enhanced motor network integration and hemispheric balance, reflected in connectivity and clinical outcomes. EEG-based machine learning tools offer scalable solutions for precision assessment of neurorehabilitation.

## Introduction

Stroke is a leading cause of long-term disability and mortality worldwide and often leads to motor and sensory impairments due to disrupted cerebral blood flow, resulting in functional limitations such as hemiparesis, impaired balance, and abnormal gait [1]. Despite standard rehabilitation, 20–30% of individuals post stroke continue to experience severe mobility challenges, making gait restoration a key therapeutic goal [2],[3],[4].

Rehabilitation efforts now emphasize intensive, repetitive, and patient-centered approaches, which are more effective at promoting neuroplasticity than traditional therapies [8, 9]. Exoskeleton-Assisted Rehabilitation (ER) has emerged as a promising strategy for improving gait and motor function in stroke survivors. ER facilitates sagittal plane movement patterns through assist-as-needed gait rehabilitation, potentially enhancing motor learning and reducing maladaptive compensation. Compared with body weight-supported treadmill training, such as the G-EO System [5] and Lokomat [6], robotic exoskeletons can allow individuals to ability to control step initiation, promote symmetry, improve midline balance and trunk control, as well as integrate the variability of visuospatial flow and vestibular stimulation. Additionally, when combined with or compared to Standard of Care (SOC) rehabilitation, comprising conventional therapist-guided gait and mobility exercises, ER offers an opportunity to maximize neural recovery and functional gains through structured progressive training [7],[8],[9]. Current literature suggests that rehabilitative interventions are more effective if they ensure early, intensive, task-specific, and multisensory stimulation, with both bottom-up and top-down integration, which favor brain plasticity [10],[11].

A key factor in optimizing rehabilitation outcomes is a deeper understanding of the neural mechanisms underlying motor recovery. Functional imaging techniques like positron emission tomography (PET) and functional magnetic resonance imaging (fMRI) have highlighted increased activation in motor and sensory cortical areas during recovery [12], [13]. However, these methods are limited by high costs and poor temporal resolution. Electroencephalography (EEG) offers a non-invasive, cost-effective, and temporally precise alternative for monitoring brain activity, making it highly suitable for clinical rehabilitation settings [14],[15], [16], [17],[18].

EEG-derived biomarkers, including power spectral changes, event-related desynchronization, and functional connectivity, have been increasingly recognized for their ability to track neural plasticity and predict recovery potential [19],[20, 21],[22, 23], [24, 25],[26, 27]. Connectivity studies can be performed during both task-based and resting-state conditions. Resting-state protocols are generally easier to implement, making them suitable even for patients in the acute phase. Notably, recent research has shown that changes in resting-state connectivity, particularly within the motor network, can predict motor function recovery in stroke patients [28],[29]. Many studies have explored how stroke affects brain resting-state connectivity, especially by comparing stroke patients in the early (acute) phase with healthy controls. However, limited studies have specifically examined how motor rehabilitation over time influences the reorganization and recovery of the brain’s motor network after a stroke [30],[31].

In particular, connectivity patterns in the alpha (8–13 Hz) and beta (14–30 Hz) frequency bands have been closely linked to motor network integrity and functional outcomes [30],[19],[9],[32]. Early post-stroke increases in motor network connectivity are often associated with better recovery trajectories, while delayed or maladaptive connectivity changes can hinder progress [33].

Despite this potential, many EEG studies remain limited to surface-level analyses, lacking source-level precision, directionality, and frequency-specific insights that are critical for understanding complex network reorganization. Connectivity computed in electrode space is often affected by the scalp volume-conduction effect, making it difficult to interpret such findings as true brain connectivity [34],[35]. In addition, previous research focused narrowly on the primary motor cortex (M1), potentially overlooking the contributions of non-motor regions and broader network dynamics essential for functional recovery [32],[36]. Recent frameworks, such as CAMBA [31], have provided valuable insights into hemispheric asymmetry dynamics following stroke by analyzing multi-level brain interactions at the sensor level/surface. However, these studies primarily focused on lateralization indices and homotopic comparisons, which may overlook the broader network dynamics crucial for functional recovery. Additionally, their reliance on scalp-level EEG limits spatial specificity, making it difficult to resolve directed interactions among cortical regions.

In contrast, the current investigation advances this field by performing a whole-brain, source-level analysis of effective connectivity using Partial Directed Coherence (PDC). This approach enables a physiologically precise characterization of directed, frequency-specific interactions across the entire cortical network, not limited to the primary motor cortex or homotopic regions. By analyzing global network reorganization, we aim to capture the contributions of both motor and non-motor regions to post-stroke recovery, a critical aspect often neglected in prior EEG connectivity studies. Furthermore, while previous studies have explored interventions such as brain-computer interface (BCI) rehabilitation, we focus on longitudinal connectivity changes following gait rehabilitation (ER and SOC programs), providing insights into how large-scale network dynamics support functional recovery in ecologically valid, mobility-related contexts. Integrating graph theory metrics and machine learning, we also investigate the potential of whole-brain connectivity patterns as biomarkers for monitoring and predicting motor recovery.

The primary aim of this study was to investigate how gait rehabilitation influences whole-brain effective connectivity patterns in individuals post stroke, with a particular focus on frequency-specific, directed interactions between cortical regions. We hypothesized that individuals post stroke would exhibit widespread disruptions in directed connectivity across both motor and non-motor networks compared to healthy controls, reflecting impaired integration of sensorimotor and associative circuits. Furthermore, we predicted that rehabilitation would promote network reorganization through enhanced connectivity in affected regions, rebalancing interhemispheric interactions and strengthening global integration. By leveraging source-level EEG and advanced network analyses, we aimed to capture these neuroplastic changes beyond traditional motor-centric models. Finally, we hypothesised that machine learning models trained on whole-brain, frequency-specific connectivity features would reliably differentiate post stroke individuals from healthy controls and detect post-rehabilitation improvements, establishing these features as sensitive, objective biomarkers of recovery potential.

## Materials and methods

### Participants

Data from this investigation is part of a larger randomized clinical trial and preliminary data is presented. Data is included for 22 healthy controls (HC) and 22 individuals diagnosed with acute ischemic or hemorrhagic stroke (mean age: 59.2 ± 9.3). All procedures performed in this investigation were approved by the Human Subjects Review Board and informed consent was obtained prior to participation in the study. Individuals diagnosed with stroke were recruited from an inpatient rehabilitation hospital, and all participants provided written informed consent in accordance with the Declaration of Helsinki. Inclusion criteria for stroke participants were: (1) age between 40 and 75 years; (2) diagnosis of unilateral hemiparesis; (3) time since stroke onset less than 40 days; 4) ability to physically fit into the exoskeleton device (height between 1.5 and 1.8m; weight < 99.7 kg); 5) joint range of motion within normal functional limits for ambulation; and 7) ability to follow directions (Mini-Mental State Examination score > 24).

Exclusion criteria included: 1) any medical issues preventing full weight bearing and ambulation; 2) skin issues that would prevent wearing the device; 3) pre-existing conditions causing exercise intolerance; 4) neuromuscular or neurological pathologies interfering with lower limb range of motion; 5) bilateral or brainstem lesions; (6) severe aphasia or cognitive impairment precluding study participation; or (6) contraindications to EEG recording (e.g., scalp wounds, implanted devices).

Healthy controls were age and sex-matched to the patient group and had no history of neurological or psychiatric illness. Demographic and clinical characteristics, including age, sex, handedness, lesion side and location, and baseline Fugl-Meyer Assessment (FMA) (lower extremity motor) scores, were recorded for all participants (see Table 2). The FMA [37] was used as a measure of lower extremity motor function and includes a 34-point motor domain scale that measures movement, coordination, and reflex action about the hip, knee, and ankle.

### Experimental Design

This study employed a single-blind, randomized controlled design to evaluate the effects of two rehabilitation interventions, Exoskeleton-Assisted Rehabilitation (ER) and Standard of Care (SOC) on neural and functional recovery post stroke. Upon enrollment, individuals diagnosed with stroke were randomly assigned (using computer-generated block randomization) to either the ER group (n = 9) or the SOC group (n = 13). Randomization was stratified by age and baseline FMA score to ensure balanced group allocation. Healthy controls did not receive any intervention but underwent the same EEG assessment protocol for baseline comparison.

Individuals diagnosed with stroke in both intervention groups (ER and SOC) participated in a 10-week gait rehabilitation program, consisting of three sessions per week (30 total sessions each session lasting 30 minutes). The ER group received gait training using a robotic exoskeleton device (EksoNR, Ekso Bionics, Inc. Richmond, CA, USA), which provided overground gait rehabilitation through task-specific stepping, with variable motor assistance as needed). The SOC group received conventional overground gait rehabilitation according to the clinical practice guidelines. Both groups focused on repetitive stepping, weight shifts and step initiation during gait rehabilitation sessions under the guidance of a licensed physical therapist. Heart rates were monitored in each group to drive the walking intensity throughout the sessions.

EEG recordings were collected for participants post stroke at two time points: (1) baseline (prior to gait intervention); and (2) post-intervention (after 10 weeks, 30 sessions of gait intervention). EEG recordings for heathy controls were collected at a single session and healthy control participants did not receive a gait intervention. All EEG assessments were performed in an instrumented gait lab, the environment was quiet, and participants seated comfortably and instructed to remain relaxed with their eyes open. Clinical assessments, including the FMA and additional functional outcome measures, were administered at baseline and post-intervention for the stroke group and at a single session for the healthy controls.

### EEG Data Acquisition

EEG data was collected while participants were in resting state. The EEG data was recorded using a 64-channel wireless ActiCap EEG system (Brain Products, Munich, Germany) equipped with active electrodes for dual-stage amplification, significantly reducing movement artifact noise. The electrode montage followed the 10–20 system for locations. Participants were asked to remain still, keep their eyes open and avoid blinking or jaw clenching during the 3-minute recording to reduce artifacts. The EEG data was collected at a sampling rate of 500 Hz, and FCz was chosen as the reference during data collection.

### EEG Data Analysis

The EEG data was preprocessed using the EEGLab toolbox [38], FieldTrip [39], and custom algorithms for further data analysis. The process began with a quality check and manual removal of artifacts. Next, the data was band-pass filtered between 1 and 50 Hz. The signal was then processed using Independent Component Analysis (ICA), which separates multivariate EEG signals into independent components (ICs), distinguishing neural activity from artifacts, followed by wavelet-ICA (wICA) [40] for further artifact suppression. Wavelet decomposition was then applied to artifact-related ICs, using biorthogonal wavelet transformations to suppress artifacts while preserving neural signals. Wavelet coefficients exceeding a threshold were set to zero, and the Inverse DWT reconstructed these ICs with the artifacts suppressed while preserving neural signals [40]. The clean EEG signals were then further smoothed using Empirical Mode Decomposition (EMD) [41], which is used to refine the cleaned signal by summing selected intrinsic mode functions (IMFs) to reconstruct artifact-free data. Finally, the EEG data were re-referenced offline to a common average reference to eliminate baseline shifts and ensure uniformity across electrodes.

To accurately estimate directed functional connectivity from EEG signals, it is crucial to address the volume conduction effect, where electrical activity propagates from the brain through conductive tissues (e.g., skull and scalp) and fluids (e.g., cerebrospinal fluid) to the scalp electrodes, as ignoring this effect can lead to misleading connectivity results. One effective way to mitigate this is to analyze source-level activity rather than scalp-level recordings. Therefore, we used the Linearly Constrained Minimum Variance (LCMV) beamforming technique, as implemented in FieldTrip [39], to reconstruct source-level signals for each condition. We employed a standard BEM (Boundary Element Method) head model, standard_1005 electrode positions, and the Brainnetome atlas to define the spatial and anatomical framework. A leadfield was computed using a 4 mm resolution grid, and LCMV beamforming was applied with a 5% regularization parameter to localize brain activity across 246 regions. These regions were then parcellated and reduced to 48 functional areas using singular value decomposition (SVD) [39]. Finally, source power was averaged across trials, resulting in a dataset of virtual channel time courses for each subject, with corresponding region labels stored for subsequent analysis. Sources from deep structure or those less affected by the stroke were excluded, resulting in 28 sources. EEG data from the localized sources were then segmented into epochs of 4 seconds, each corresponding to 2000 datapoints. The total number of epochs for each subject and condition was set equally to 29 epochs. Thus, we have a 3D matrix that is 28*2000*29, corresponding to the number of brain sources localized, segmented data points and number of epochs. Since subjects had injuries in different injured hemispheres, EEG data from patients with lesions in their right hemisphere were flipped to enable consistency throughout the data analysis process, which allowed us to uniformly define the left hemisphere as the affected side and the right hemisphere as the unaffected side. The clean EEG data was then decomposed into five frequency bands: delta (0.1–4 Hz), theta (4–8 Hz), alpha (8–13 Hz), beta (13–30 Hz) and gamma (30–50 Hz). Finally, we estimated the directed functional connectivity networks (FCN) for each frequency band using partial directed coherence (PDC), and estimated the local efficiency, node degree and node strength as described in previous studies [42],[43],[44]. For each PDC map, we performed automated matrix binarization, using the orthogonal minimum spanning trees method [45], to select the true connections between the EEG channels. The resulting binarized PDC maps and graph analysis measures were used for further analysis.

### Correlation Analysis and Laterality Index (LI)

The study conducted bivariate correlation analysis to examine the relationship between post-stroke changes in EEG functional connectivity and motor recovery measured by the Fugl-Myer (FMA). We subtracted the baseline (pre-intervention) score from the post-intervention score for both the FMA and EEG connectivity within each hemisphere and plotted their correlation. Positive correlation suggests that greater increases in EEG connectivity are associated with better motor recovery. The lateralization of functional connectivity for each brain source region was evaluated using the laterality index (LI), calculated as LI = (Unaffected - Affected) / (Unaffected + Affected), where affected represents the estimated outward connectivity values for the area of the lesion. Positive LI values indicate unaffected dominance, while negative values denote affected dominance, and values close to 0 referred to symmetrical connectivity. Only LI distributions with mean values significantly different from zero, at a threshold of p < 0.05, were considered to demonstrate statistically significant lateralization toward either hemisphere.

### Statistical Analysis and Machine Learning Classification

Statistical analyses and machine learning were developed and executed using MATLAB 2024a (The MathWorks, Inc., Natick, Massachusetts, United States). Normality of the data were checked with a Shapiro Wilk test, and where required, nonparametric statistics were applied. Within-group comparisons were performed using 2-sided paired t tests and across-group comparisons using 2-sided unpaired t tests. Three testing comparisons were established, Stroke Post intervention vs Stroke Pre intervention, Healthy Control vs Stroke Pre intervention and Healthy Control vs Stroke Post intervention. A p-value of 0.01 (p = 0.01) was considered statistically significant following Keppel modified Bonferroni criteria [46], thus reducing the risk of Type I error and increasing the rigor of the statistical inference. This comprehensive approach allowed for a robust analysis of the connectivity differences between conditions and groups, as well as the evaluation of hemispheric lateralization. For each test, the effect sizes were reported using Cohen’s d (d).

After completing the statistical analysis, we conducted binary classification across three group comparisons: Healthy Controls vs. Stroke Pre intervention, Healthy Controls vs. Stroke Post intervention, and Stroke Pre intervention vs. Stroke Post intervention. A Support Vector Machine (SVM) classifier with a Radial Basis Function (RBF) kernel was used for this study. The full description of the classifier can be found in our previous work [42],[47]. Classification was performed separately for each frequency band (delta, theta, alpha, beta, and gamma). To prepare the data, connectivity matrices were flattened into feature vectors, and features were z-score normalized to ensure equal scaling. To reduce dimensionality and enhance model performance, the Relief algorithm [48] was employed for feature selection. SVM hyperparameters were optimized via 5-fold cross-validation grid search (C: [0.1, 1, 10], kernel scale: [0.01, 0.1, 1]) with the best combination (C = 1, kernel scale = auto) selected based on classification accuracy. The SVM model was evaluated using leave-one-out cross-validation (LOOCV), where the model is trained on all subjects except one, and then tested on the excluded subject. This process is repeated for each subject. To mitigate overfitting, we ensured feature selection was performed within each LOOCV fold. Model performance was assessed using four standard metrics: accuracy, sensitivity, specificity, and the area under the Receiver Operating Characteristic (ROC) curve.

## Results

### Clinical Scales Results:

**Figure 1:**
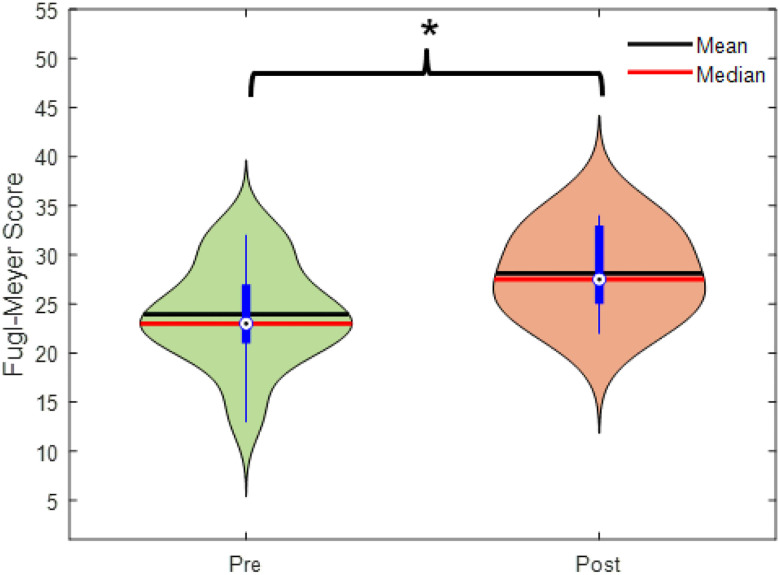
Fugl-Meyer score for all participants post stroke at baseline (pre intervention) and post intervention (after 10 weeks and 30 gait training sessions).

Figure 1 presents the distribution of Fugl-Meyer Assessment (FMA) scores before and after the 10-week intervention for all individuals post stroke, regardless of rehabilitation group (ER or SOC). The mean FMA score improved significantly across all participants, increasing from 23.95 ± 5.01 (pre-intervention) to 28.13 ± 4.08 (post-intervention) (*t*(21) = 2.612, *p* = 1.70 × 10^−5^, Cohen’s *d* = 1.181), indicating robust motor recovery following the rehabilitation program. A two-way mixed ANOVA was conducted to assess the effects of group (ER, n = 9; SOC, n = 13) and time (pre vs. post-intervention) on FMA scores. The analysis revealed no significant main effect of group (F(1, 20) = 0.19, p = 0.665) or Group × Time interaction (F(1, 20) = 0.002, p = 0.9875). These results indicate comparable motor improvements in both ER (mean increase: 4.33 ± 3.91) and SOC (mean increase: 4.08 ± 2.93) in this preliminary dataset. These results may change as we increase our sample for both ER and SOC groups. Given the absence of significant group differences in motor improvement between ER and SOC interventions (p > 0.65), subsequent neural analyses were performed on the pooled patient cohort to maximize statistical power and focus on shared neuroplastic mechanisms underlying motor recovery.

### Comparison of the directed functional connectivity

For the purpose of this study, we focused our analysis on the alpha frequency band which has been reported as the most relevant for motor system functions [49]. Results for the other frequency bands are provided in the supplementary materials (Fig. 1–10).

### Between-Group Comparisons:

#### HC vs Stroke Baseline (Pre-Intervention)

**Figure 2:**
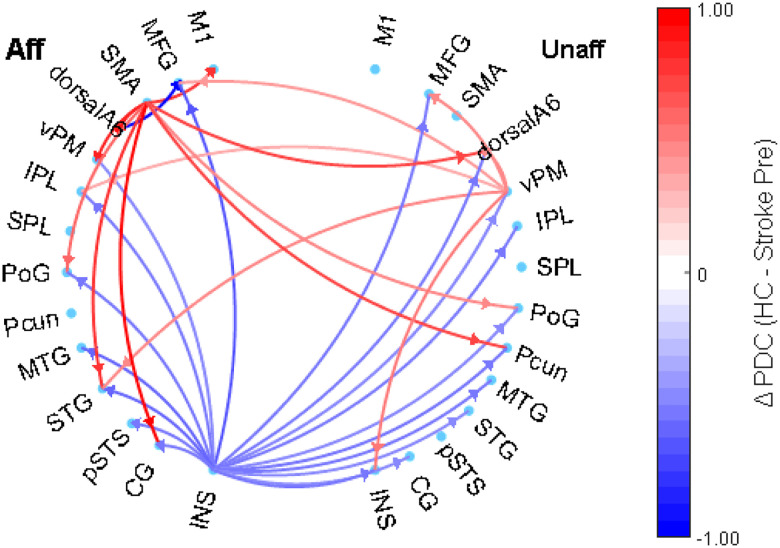
Directed connectivity network between healthy control (red lines) vs an individual post stroke pre intervention (blue lines).

Figure 2 shows group-level differences in the alpha band between healthy controls (HC) and stroke patients before the intervention. Red arrows denote stronger connectivity in HC; blue arrows indicate stronger connectivity in stroke patients. HC showed dominant outgoing connections from the SMA in the left hemisphere toward PoG (t(42) = 2.77, p = 0.0083) and dorsalA6 (t(21) = 3.58, p = 8.6×10^−4^) in both hemispheres, and towards the vPM (t(21) = 3.12, p = 0.0032) in the left hemisphere, indicating robust motor network integration. The right vPM also exhibited elevated connectivity in HC (t(42) = 2.76, p = 0.01).

Conversely, individuals post stroke before intervention demonstrated enhanced connectivity from the insula (INS) in the affected hemisphere (t(21) = − 3.12, p = 0.0032) towards various frontal and parietal areas, as well as from the affected dorsalA6 to frontal regions (t(21) = − 2.79, p = 0.0077), likely reflecting compensatory recruitment. Overall, these results suggest that the HC group maintained more cohesive motor network organization, while the baseline (pre intervention) stroke group relied on alternative pathways.

#### HC vs Stroke Post-Intervention (after 30 sessions)

**Figure 3:**
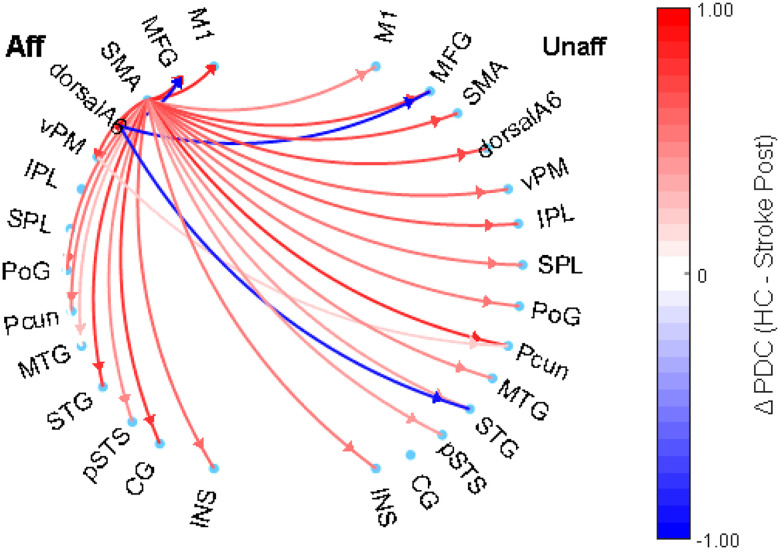
Directed connectivity network between healthy control (red lines) vs Individual post stroke patient post intervention (blue lines).

In Fig. 3, red arrows show widespread stronger connectivity in HC from the SMA within the affected hemisphere toward multiple regions in both hemispheres (2.7 < t(42) < 4.7, p < 0.008). In contrast, the few blue arrows from dorsalA6 towards MFG in both hemispheres (t(42) < − 3, p = 0.004) and STG in the unaffected region (t(42) < − 2.8, p = 0.007) suggest improved connectivity for individuals post stroke post-intervention. These findings indicate that although individuals post stroke exhibit limited network integration compared to HCs, the observed changes may reflect targeted compensatory mechanisms post-intervention.

### Within Stroke Group:

**Figure 4:**
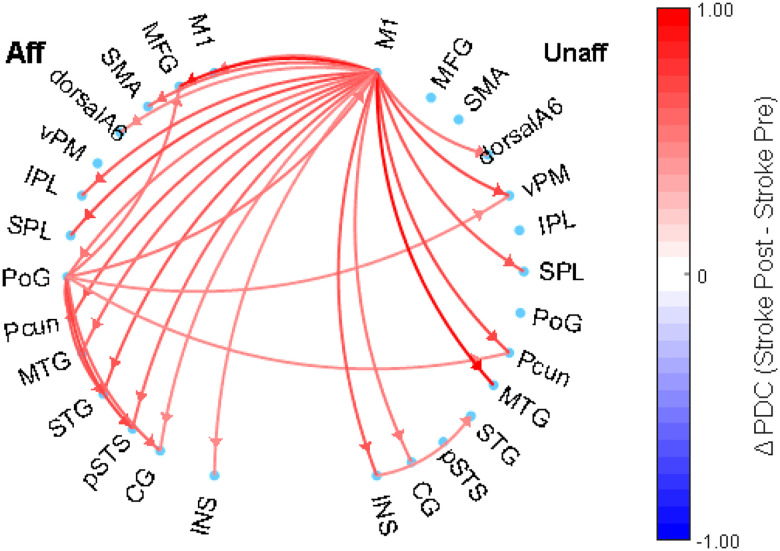
Directed connectivity network between an individual post stroke: post intervention vs baseline (pre intervention).

Figure 4 highlights statistically significant changes in directed functional connectivity within the alpha band. Notably, we observed enhanced outgoing connectivity from the Primary Motor Cortex (M1) in the unaffected hemisphere to several cortical regions, including the middle temporal gyrus (MTG; t(21) = 2.97, p = 0.0073), precuneus (Pcun; t(21) = 3.14, p = 0.0049), postcentral gyrus (PoG; t(21) = 2.82, p = 0.013), supplementary motor area (SMA; t(21) = 2.74, p = 0.012), and dorsal area 6 (dorsalA6; t(21) = 2.7, p = 0.013).

Similarly, the affected hemisphere exhibited elevated directed connectivity from the PoG toward Pcun (t(21) = 2.91, p = 0.0083), superior temporal gyrus (STG; t(21) = 2.83, p = 0.01), M1 (t(21) = 2.7, p = 0.013), middle frontal gyrus (MFG; t(21) = 2.98, p = 0.0070), and ventral premotor cortex (vPM; t(21) = 2.74, p = 0.012). This pattern indicates a post-intervention increase in motor-related communication across multiple cortical networks, which is suggestive of neuroplastic reorganization and functional recovery.

### Graph Network Analysis

In contrast to the statistically thresholded difference maps presented earlier, the graph network measures used, which include **node strength**, **local efficiency**, and **node degree**, were calculated directly from the directed functional connectivity maps for each group (Healthy Controls, Stroke Group Baseline Before Intervention, and Stroke Group Post-Intervention). These metrics offer insights into the intrinsic topological organization of the brain networks under each condition.

#### Node Strength

**Figure 5:**
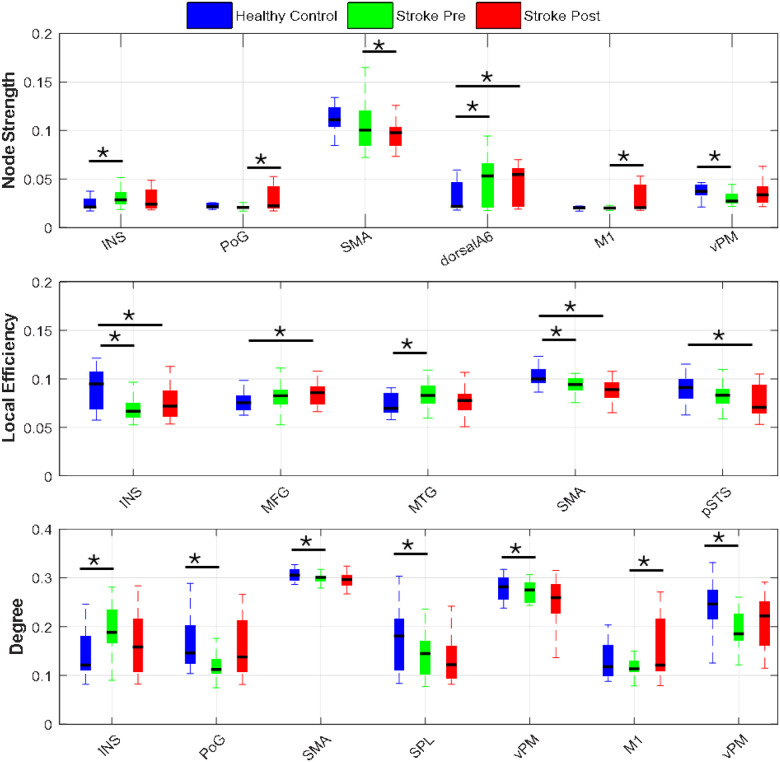
Graph network metrics (node strength, local efficiency, node degree) across Healthy Controls and Stroke survivors Pre- and Post-Intervention.

Figure 5 shows the graph network metrics (node strength, local efficiency, node degree) across the HC group and the stroke group at baseline and post intervention. Significant differences in node strength were observed between groups and across brain regions. HC exhibited higher strength in the ventral premotor cortex (vPM) of the right hemisphere compared to the stroke group before intervention (t(42) = 2.67, p = 0.0106, Cohen’s d = −0.807). In contrast, the stroke group demonstrated elevated node strength in the insula (IN) (t(42) = −2.5, p = 0.0166, Cohen’s d = 0.752) and dorsal area 6 (dorsalA6) (t(42) = −2.57, p = 0.0139, Cohen’s d = 0.774) of the affected hemisphere, suggesting region-specific compensatory mechanisms.

Following intervention, dorsalA6 in individuals post stroke exhibited a further increase in node strength compared to HC (t(42) = −2.81, p = 0.0074, Cohen’s d = 0.848), while SMA in the affected hemisphere showed a significant decrease (t(42) = 2.61, p = 0.0124, Cohen’s d = −0.788). Within-group comparisons showed post-intervention increases in node strength in the postcentral gyrus (PoG) of the affected hemisphere (t(21) = 2.86, p = 0.0093, Cohen’s d = 0.61) and in the primary motor cortex (M1) of the unaffected hemisphere (t(21) = 3.25, p = 0.0064, Cohen’s d = 0.645), reflecting enhanced integration in motor-related networks post intervention.

#### Local Efficiency

As shown in the group-level comparisons, the stroke group demonstrated significantly reduced local efficiency as compared to the HC group prior to intervention, particularly in the insula (INS) (t(42) = 3.523, p = 0.001, Cohen’s d = −1.062) and supplementary motor area (SMA) (t(42) = 3.029, p = 0.0042, Cohen’s d = −0.913) of the affected hemisphere. Interestingly, an increase was observed in the middle temporal gyrus (MTG) (t(42) = −2.814, p = 0.0074, Cohen’s d = 0.848), potentially indicating network-level adaptation.

Post intervention, individuals in the stroke group continued to show decreased local efficiency in the SMA (t(42) = 3.948, p = 2.9×10^−4^, Cohen’s d = −1.19) and INS (t(42) = 2.56, p = 0.0141, Cohen’s d = −0.772) compared to HC, along with a decrease in the posterior superior temporal sulcus (pSTS) of the unaffected hemisphere (t(42) = 2.69, p = 0.01, Cohen’s d = −0.814), and an increase in the middle frontal gyrus (MFG) (t(42) = −2.55, p = 0.0144, Cohen’s d = 0.77). However, no significant changes were observed within the stroke group when comparing pre and post intervention efficiency.

#### Node Degree

Before the intervention, individuals post stroke exhibited a reduced node degree in the postcentral gyrus (PoG) of the affected hemisphere (t(42) = 2.55, p = 0.0146, Cohen’s d = −0.768) and in the vPM of the unaffected hemisphere (t(42) = 3.562, p = 9.3×10^−4^, Cohen’s d = −1.074), indicating disrupted integration in key motor and sensorimotor regions. Conversely, the insula (INS) in the affected hemisphere showed increased node degree (t(42) = −3.248, p = 0.0023, Cohen’s d = 0.979), consistent with a possible shift toward localized compensatory processing.

After intervention, node degree remained significantly lower for individuals post stroke in the affected hemisphere, particularly in the vPM (t(42) = 2.6, p = 0.013, Cohen’s d = −0.783), SMA (t(42) = 2.6, p = 0.0129, Cohen’s d = −0.783), and superior parietal lobule (SPL) (t(42) = 2.52, p = 0.0157, Cohen’s d = −0.759). However, within-group comparisons revealed a post-intervention increase in node degree in the M1 of the unaffected hemisphere (t(21) = 2.66, p = 0.0145, Cohen’s d = 0.568), aligning with trends observed in node strength and suggesting partial network recovery.

### Correlation analysis and laterality

#### Correlation Analysis:

**Figure 6:**
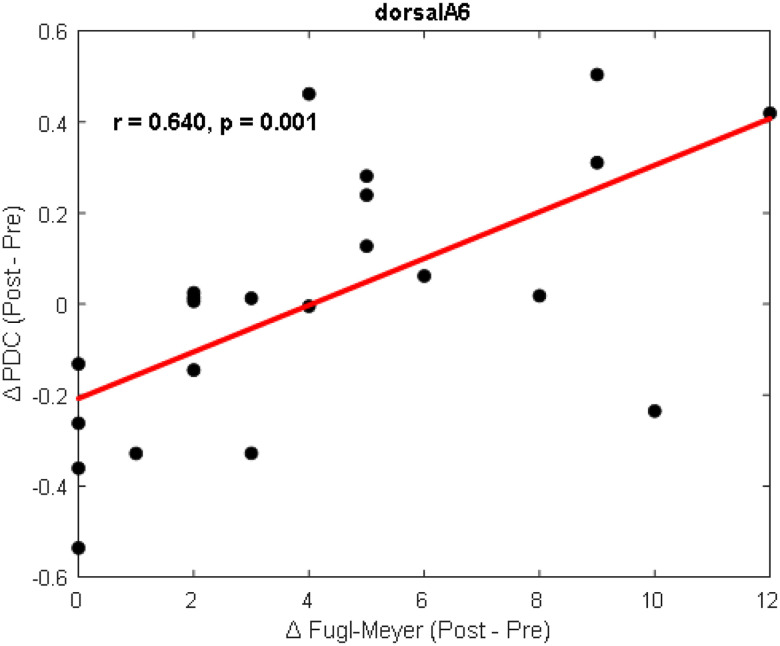
Correlation analysis between connectivity and Fugl-Myer at dorsalA6 area.

Figure 6 shows the relationship between EEG-derived functional connectivity and motor function represented by the FMA score. Among the various brain regions analyzed, only the dorsalA6 region in the affected hemisphere demonstrated a statistically significant correlation. Specifically, increased cortical connectivity in dorsalA6 was positively associated with higher Fugl-Meyer scores (r = 0.640, p = 0.001), suggesting that stronger connectivity in this motor planning region may serve as a reliable neural marker for improved motor performance in stroke survivors. This finding emphasizes the role of dorsal premotor areas in motor recovery and highlights their potential use in clinical monitoring and intervention planning.

#### Changes in the Laterality Index:

**Figure 7:**
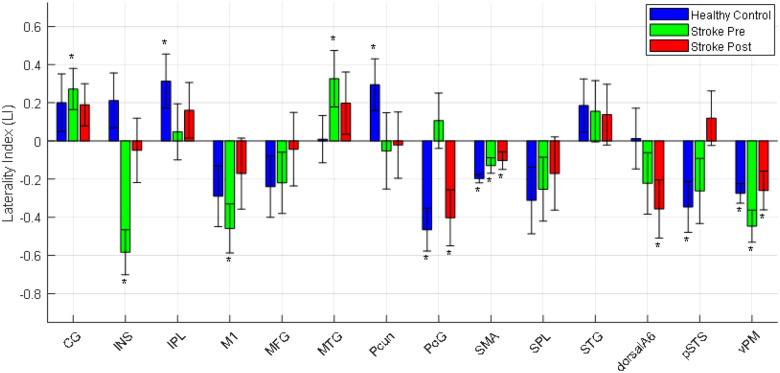
Laterality Index in different brain cortices in healthy control and stroke survivors pre and post intervention.

Figure 7 displays the Laterality Index (LI) values for Healthy Controls (HC), Stroke Baseline (before intervention), and Stroke-Post intervention groups. The LI quantifies hemispheric asymmetry: in healthy individuals, positive LI values indicate right hemispheric dominance, while negative values indicate left dominance. For individuals post stroke, positive LI values represent dominance in the unaffected hemisphere, and negative values reflect dominance in the affected hemisphere. Asterisks (*) denote statistically significant lateralization from zero (p < 0.05), highlighting meaningful inter-hemispheric differences.

Among healthy individuals, significant right-hemispheric dominance was observed in the Inferior Parietal Lobule (IPL) (p = 0.037) and Precuneus (Pcun) (p = 0.040), while left dominance was found in the Postcentral Gyrus (PoG) (p = 4.5×10^−4^), Supplementary Motor Area (SMA) (p = 1.5×10 − 8), posterior Superior Temporal Sulcus (pSTS) (p = 0.017), and ventral Premotor Cortex (vPM) (p = 2.8×10 − 5).

In the Stroke-Baseline (before intervention) group, exaggerated lateralization was evident:

- Unaffected hemisphere dominance was seen in the Cingulate Gyrus (CG) (p = 0.0198) and Middle Temporal Gyrus (MTG) (p = 0.0386).
- Affected hemisphere dominance was significant in the Insula (INS) (p = 6.9×10^− 5^), Primary Motor Cortex (M1) (p = 0.0018), SMA (p = 0.005), and vPM (p = 2.8×10^− 5^).

Post-intervention, the data revealed patterns of lateralization normalization:

- Exaggerated dominance in the CG and MTG (unaffected hemisphere) was reduced.
- In the M1 (affected hemisphere), lateralization shifted toward balance, mirroring the healthy control pattern.
- The PoG showed a significant shift (p = 0.0119) from unaffected to affected hemisphere dominance, indicating recovery.
- The INS exhibited reduced affected-side dominance, while in pSTS, lateralization shifted toward the unaffected hemisphere, suggesting functional reorganization.
- These findings highlight the Laterality Index as a sensitive marker of post-stroke neuroplasticity, demonstrating region-specific recovery and adaptive changes following rehabilitation.

### Classification

**Figure 8:**
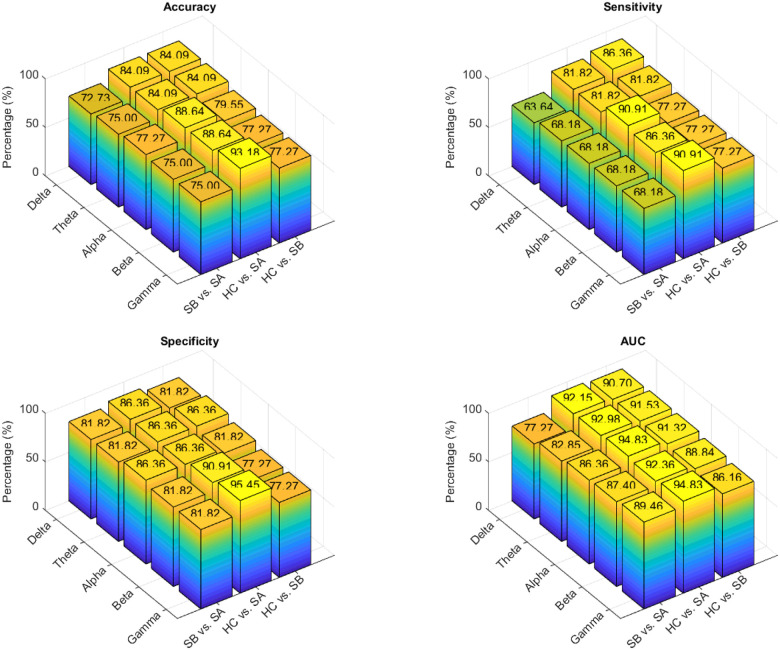
Classification Performance evaluation for the three groups: (Stroke Bassline [SB] vs. Stroke Post Intervention [SA], HC vs. SA, HC vs. SB).

Figure 8 presents classification performance across five EEG frequency bands (Delta, Theta, Alpha, Beta, Gamma) for three group comparisons (Stroke Baseline (Before Intervention) [SB] vs. Stroke Post Intervention [SA], HC vs. SB and HC vs. SA). Performance is evaluated using four key metrics: Accuracy, Sensitivity, Specificity, and Area Under the Curve (AUC).

#### Accuracy

The highest classification accuracy was observed for the HC vs. SA comparison in the Gamma band (93.18%), followed closely by the Alpha and Beta bands (88.64%). For HC vs. SB, the best performance was achieved in the Delta and Theta bands (84.09%), with the Alpha band also performing well at 79.55%. In distinguishing between SB vs. SA, the Alpha band yielded the highest accuracy at 77.27%, suggesting it is particularly sensitive to changes following stroke intervention.

#### Sensitivity

The system’s ability to correctly identify positive cases was strongest in the Alpha and Gamma bands for HC vs. SA, both peaking at 90.91%. This indicates that these bands are highly responsive to post-stroke neural dynamics and serve as effective indicators of intervention outcomes.

#### Specificity

The Gamma band demonstrated the highest specificity at 95.45% for HC vs. SA, followed by the Beta band at 90.91%, underscoring their strength in accurately detecting healthy individuals. The Alpha band also showed strong performance in differentiating stroke stages, reaching 86.36% specificity for SB vs. SA.

#### AUC (Area Under the Curve)

The most robust AUC values were recorded in the Alpha and Gamma bands for HC vs. SA (both at 94.83%), while Theta (91.53%) and Alpha (91.32%) bands also showed strong discriminative power in the HC vs. SB comparison. These high AUC scores highlight the overall classification reliability of these bands.

## Discussion

This study examined resting-state EEG source-level functional connectivity in individuals post stroke before and after a 10-week rehabilitation program and a comparative group of HCs. Unlike many previous EEG-based studies, which often focus on a limited set of regions, we estimated directed functional connectivity across the whole-brain source space. This approach offers increased reliability by reducing the influence of volume conduction artifacts, thereby enabling more accurate assessment of true neural interactions. While many prior studies focus primarily on the primary motor cortex (M1) and its immediate connections, often guided by a priori assumptions, such a narrow scope may overlook critical changes in non-motor regions that contribute to recovery. Our study challenges this convention by adopting a whole-brain connectome approach, allowing us to explore broader network dynamics, including regions not traditionally associated with motor control but which may play compensatory or integrative roles during recovery. We further investigated laterality patterns and machine learning-based classification to capture subtle and distributed changes in brain organization post-stroke. By expanding the analysis beyond M1 and incorporating directed connectivity, laterality indices, and graph network metrics, we identified a range of cortical contributors to motor recovery including regions like dorsalA6, vPM, SMA, and INS that would likely be missed using a restricted regional approach. Overall, our findings offer novel insights into the neuroplastic mechanisms underlying post-stroke motor recovery. They demonstrate the value of whole-brain, source-level EEG connectivity combined with data-driven methods. The results support the use of EEG-based connectivity features and machine learning as promising, non-invasive biomarkers for tracking rehabilitation outcomes and guiding individualized therapy strategies.

### Functional Connectivity Changes and Neuroplasticity

Our findings showed that functional connectivity was significantly decreased in individuals with subacute stroke prior to intervention compared to HCs, particularly within the affected hemisphere at the supplementary motor area (SMA) and within the unaffected hemisphere at the ventral premotor cortex (vPM). Although this preliminary investigation included two rehabilitation protocols: ER and SOC, no significant differences in clinical motor recovery were observed between groups, as indicated by the lack of a Group × Time interaction effect on FMA scores, though this was expected a larger sample may be required to detect a difference in recovery between groups. This suggests that both interventions induced comparable lower extremity motor functional improvements within the studied timeframe. Our findings align with previous research indicating that stroke disrupts functional communication between brain regions, leading to reduced neural efficiency and impaired motor control [50],[30],[51],[52],[53]. In contrast, we observed a significant increase in connectivity (t(21) = −3.12, p = 0.0032) within the affected insula (INS) projecting to both hemispheres, as well as a modest increase in dorsal area 6 (dorsalA6). These patterns reflect the INS role in interoception and cognitive regulation and mirror earlier findings suggesting that the INS plays a compensatory role in motor network deficits [52],[54],[55]. The recruitment of such regions, predominantly within the contralesional hemisphere, likely reflects an adaptive mechanism to compensate for damage to primary motor networks [56]. Nevertheless, the subsequent increase in connectivity in a limited number of brain regions does not seem sufficient to avoid a drastic reduction in the information propagation in the functional networks of the patient’s brain.

Meanwhile, after 10 weeks of rehabilitation the connectivity from dorsalA6 in the affected hemisphere increased further, extending to bilateral cortical regions. This sustained and expanded activation suggests that dorsalA6 may serve as a core hub for motor reorganization and independent compensation, facilitating recovery. Nevertheless, the connectivity network at SMA of the affected hemisphere remain higher in HC compared to individuals post stroke post-intervention. One interesting finding is that the connectivity in the INS of the affected hemisphere and the vPM in the unaffected hemisphere normalized to levels similar to HC, implying a reduction in reliance on compensatory connections as motor function recover progressed following 10 weeks of rehabilitation.

The within-group analysis for the stroke group revealed that after 10-weeks of rehabilitation there were significant improvements in functional connectivity, reflecting neuroplastic changes associated with motor recovery. Notably, there was a marked increase in directed connectivity from the primary motor cortex (M1) in the unaffected hemisphere toward both hemispheres, suggesting strengthened interhemispheric communication, which is a recognized mechanism supporting functional compensation and recovery following stroke. In parallel, the postcentral gyrus (PoG) in the affected hemisphere exhibited increased outgoing connectivity, indicating a re-engagement of sensory-motor integration pathways that are often disrupted post-stroke. Previous studies have shown that the increased connectivity with the contralesional cerebellum after intervention could be related to the ongoing relearning of motor skills induced by the rehabilitation program [57], [50]. Importantly, these neural changes were accompanied by significant behavioral improvements, as evidenced by increased FMA lower extremity motor scores after the intervention (mean increase: 4.18 ± 3.29, *p* = 1.70 × 10^−5^, *d* = 1.18), supporting the efficacy of both ER and SOC interventions. Of particular interest, we observed that increased connectivity in dorsalA6, a region implicated in motor planning and execution, was positively associated with motor recovery, especially within the affected hemisphere (*r* = 0.640, *p* = 0.001). This finding aligns with existing literature suggesting that rehabilitation-induced neuroplasticity manifests as strengthened integration within cortical motor networks, supporting the recovery of voluntary motor function [58], [9]. Together, our results highlight the efficacy of intensive rehabilitation in promoting functional reorganization within the brain’s motor systems. They also underscore the value of directed functional connectivity analysis as a sensitive neurophysiological marker for tracking recovery and evaluating the impact of targeted interventions.

To further understand the topological reorganization of the brain networks, our graph theory analysis measures revealed distinct structural and functional changes across time. Before intervention, individuals post stroke showed lower node degree and local efficiency in key motor areas, including the SMA and PoG, reflecting reduced processing efficiency and network disruption. In contrast, node strength was abnormally elevated in the INS and dorsalA6, likely indicating early-stage hyperconnectivity as a compensatory response. Following the intervention, these network properties changed notably: dorsalA6 exhibited significant increases in node strength and degree, reinforcing its role as a reorganized motor hub, while vPM and SMA connectivity measures in individuals post stroke begin to resemble those of HC, consistent with functional normalization.

In summary, our results demonstrate that stroke recovery is characterized by a dynamic reorganization of brain networks, particularly within motor and associative regions. The integration of directed functional connectivity, graph theory measures, and behavioral correlations provides a comprehensive understanding of post-stroke neuroplasticity. These insights support the potential of non-invasive EEG-based monitoring tools as scalable, cost-effective biomarkers for evaluating treatment efficacy and guiding personalized neurorehabilitation strategies.

### LI Changes Between Individuals Post Stroke Pre and Post Intervention and representative HCs

Our analysis of Laterality Index (LI) provided critical insight into the hemispheric balance of functional connectivity across the HC and stroke groups both before and after intervention. In healthy individuals, LI values were generally balanced, reflecting well-distributed interhemispheric communication, especially across motor-related regions such as the M1, MFG, MTG, dorsalA6, and INS. This symmetry aligns with normative patterns of cortical organization and efficient sensorimotor integration. Our findings are consistent with previous studies showing that activity in healthy right-handed individuals is typically bilateral or slightly right-lateralized [59], [60].

In contrast, individuals post stroke at baseline (prior to intervention) exhibited a pronounced asymmetry in LI, particularly within the affected hemisphere, where connectivity was either diminished or abnormally enhanced in certain regions. Specifically, areas such as the CG and MTG demonstrated compensatory overactivation in the contralesional (unaffected) hemisphere, possibly due to its involvement in attention and error monitoring during impaired motor tasks. The INS and M1 also showed overactivation in the affected hemisphere.

Following the 10-week gait rehabilitation program, individuals diagnosed with stroke exhibited notable restoration of hemispheric balance in several regions. The CG, and MTG in the unaffected hemisphere, which had previously shown hyperactivation, returned to a more balanced LI value, suggesting a reduction in compensatory activity as the affected hemisphere regained function. Similarly, M1, INL and MFG in the affected hemisphere showed a shift toward normalized LI values, indicating increased involvement of the affected hemisphere and re-engagement of native motor pathways. These results align with previous studies showing a strong association between motor recovery and a reduction in interhemispheric connectivity between primary motor regions [61], [62], though this connectivity reduction has been reported to vary depending on the extent of motor recovery [63].

Overall, the normalization in LI measures aligns with the behavioral gains observed in FMA scores, reinforcing the notion that restoration of interhemispheric balance is a critical marker of successful stroke rehabilitation. These findings highlight the utility of LI as a sensitive and interpretable metric for evaluating hemispheric involvement during recovery and underscore the importance of designing interventions that promote re-engagement of the affected hemisphere while gradually reducing compensatory load from the contralesional side.

### Machine Learning and Frequency Specific Neural Features Classification Performance

Our machine learning analysis demonstrated that classification performance varied significantly across EEG frequency bands. This emphasizes the critical role of frequency-specific neural features in detecting stroke-related changes and recovery processes. Alpha and gamma bands consistently yielded the highest classification performance among the five frequency bands.

The alpha band outperformed other bands in distinguishing pre- versus post-intervention stroke states (labelled by: SB vs. SA in Figure.8) with mean accuracy of 77.27% and specificity of 86.36% and showed high sensitivity to motor recovery. The alpha band also produced a higher AUC of 91.32% in differentiating between HC vs SB. Both alpha and gamma achieved the highest sensitivity in AUC with an average of 90.91% and 94.83% respectively. Alpha oscillations are widely implicated in functional inhibition, attentional modulation, and sensorimotor integration, and the observed modulation of alpha connectivity likely reflects the dynamic rebalancing of cortical networks following rehabilitation. Moreover, the alpha band’s robust performance in HC versus post-intervention stroke (HC vs. SA) and pre- versus post-intervention stroke comparisons underscore its role as a neural signature of functional recovery and improved inter-regional communication.

Similarly, the gamma band, associated with high-frequency oscillatory activity critical for sensorimotor integration and higher-order cognitive processing [64], demonstrated outstanding specificity (95.45%) and sensitivity (90.91%), particularly in differentiating HC from individuals post stroke post-intervention. This suggests that stroke recovery may involve compensatory enhancements or reorganization in fast cortical rhythms, which are effectively captured within the gamma range.

The beta band, which is traditionally linked to motor control and active cognitive engagement [65], [66] showed consistent performance across all evaluation metrics ranging from 68.18–92.36%, across all group comparisons. This highlights beta-band network connectivity as a highly sensitive biomarker for stroke-induced disruptions and rehabilitation-driven neuroplasticity. The higher classification performance of the beta band across conditions reinforces their utility as reliable indicators of motor system integrity and recovery progression. Our findings are consistent with a previous EEG resting-state stroke studies, which produced 73.20% accuracy in differentiating between HC and stroke groups [64].

In contrast, the lower-frequency delta and theta bands, which are generally associated with global brain states such as fatigue or reduced vigilance and are less specific to motor system dynamics, showed moderate to lower classification performance. Nonetheless, in certain comparisons (e.g., HC vs. Stroke Before / SB), delta and theta features contributed valuable information, potentially reflecting generalized cortical slowing or diffuse dysfunction in stroke-affected individuals. Previous studies have found that lower frequency oscillations ,such as delta and theta frequencies may be useful biomarker biomarkers of stroke and may reflect both injury and recovery post stroke [63],[67].

Importantly, the frequency-specific classification results align closely with our directed connectivity and graph theory analyses, validating the fact that functional reorganization after stroke is inherently frequency-dependent. The enhanced classification accuracy observed post-intervention in the stroke group confirms that EEG-based machine learning approaches can detect subtle neurophysiological changes, supporting their practical application as objective tools for monitoring brain recovery. Together, these findings underscore the importance of leveraging frequency-specific EEG features in developing sensitive, non-invasive biomarkers for stroke rehabilitation.

### Limitations and Future Directions

While this preliminary investigation provides valuable insights into stroke recovery through resting-state EEG connectivity analysis, several limitations warrant consideration. First, although the sample size of 44 participants was determined through rigorous power analysis, it remains relatively modest, potentially limiting the generalizability of our findings to broader stroke populations and the robustness of our machine learning model. We will continue to enroll additional participants in the active clinical trial (NCT04309305), and we will aim to recruit larger cohorts for future studies to enhance statistical power and enable subgroup analyses, such as stratification by lesion location or severity. Second, our focus on subacute stroke with unilateral lesions restricts the applicability of our results to other clinical subgroups. Recovery dynamics and neural reorganization may differ substantially in chronic stroke cases or individuals with bilateral brain involvement. Expanding future research to include these populations will be critical for developing more comprehensive rehabilitation models. Third, the exclusive use of resting-state EEG, while advantageous for its simplicity and clinical feasibility, limits our understanding of task-specific neural dynamics that are essential for motor control and functional recovery. Incorporating task-based EEG paradigms or simultaneous EEG-EMG recordings could provide richer information on sensorimotor integration and cortical excitability during movement. Moreover, EEG alone offers limited spatial resolution and may be influenced by volume conduction effects despite source localization efforts. Multimodal neuroimaging approaches, such as combining EEG with functional and structural magnetic resonance imaging (MRI), or electromyography (EMG) could elucidate complementary aspects of neural plasticity and motor network reorganization. Additionally, although participants underwent two distinct rehabilitation protocols: ER and SOC, they were analyzed collectively as a single cohort. This limits our ability to discern the specific neural impact of each gait training modality. Increasing the sample size in future studies would allow for stratified analyses and a clearer understanding of how different rehabilitation strategies uniquely influence brain connectivity and recovery patterns. Finally, our study examined neural changes over a 10-week intervention period, but the long-term stability and clinical relevance of these connectivity alterations remain unclear. Longitudinal follow-up studies are needed to assess the durability of neurophysiological improvements and their predictive value for sustained functional outcomes.

## Conclusion

This study provides a comprehensive investigation into stroke-induced brain network reorganization using source-level resting-state EEG, graph theory metrics, laterality indices (LI), and machine learning across frequency specific bands. We found that stroke significantly disrupted directed functional connectivity within key motor and cognitive regions, most notably the **SMA** and **vPM**, prior to intervention. However, after a 10-week targeted rehabilitation program, connectivity from the **M1** in the unaffected hemisphere significantly increased toward bilateral cortical areas, while the **PoG** in the affected hemisphere exhibited enhanced outgoing connections. These connectivity changes imply strengthened interhemispheric communication and restored sensory-motor integration. Quantitatively, post-intervention improvements were reflected in significantly higher **FMA** scores (mean increase: 4.18 ± 3.29, *p* = 1.70 × 10^−5^, Cohen’s *d* = 1.18) and were strongly correlated with enhanced connectivity in motor-related areas, particularly **dorsalA6** (*r* = 0.640, *p* = 0.001), underscoring its role in motor planning and recovery. Graph theory metrics confirmed a shift from a disrupted and inefficient network toward a more integrated and functional topology. Notably, **node strength and degree** significantly increased in dorsalA6 and M1 post-intervention, while **local efficiency** normalized in regions such as the **MFG** and **INS**. LI analysis revealed that hemispheric asymmetry, exaggerated in post stroke at baseline (before intervention), especially in the **INS**, **CG**, and **MTG** tended toward restored symmetry after rehabilitation training, with several regions, including the **M1**, **PoG**, and **pSTS**, reestablishing interhemispheric balance. Machine learning analysis further validated these findings: classification performance between the HC and post-intervention stroke group peaked at **93.18% accuracy** and **94.83% AUC** in the **gamma band**, while **alpha band** features provided the highest accuracy (**77.27%**) for distinguishing between pre- and post-intervention stroke states. These results highlight alpha and gamma oscillations as critical markers of recovery-related neuroplasticity.

## Supplementary material

Supplementary materials are available in a separate file.

Supplementary Files

This is a list of supplementary files associated with this preprint. Click to download.

- Supplementarymaterialsforstrokepaper.pptx

## Figures and Tables

**Table 1:** List of abbreviations used in the paper

Abbreviation	Description	Abbreviation	Description
HC	Healthy controls	CG	Cingulate Gyrus
CNS	Central nervous system	INS	Insula
Pre	Baseline, before gait intervention (stroke and HC group)	IPL	Inferior Parietal Lobule
Post	Post Intervention (after 30 sessions) (stroke group only)	M1	Primary Motor Cortex
Unaff	Brain region/hemisphere Unaffected by stroke	MFG	Middle Frontal Gyrus
Aff	Brain region Affected by stroke (lesion side)	MTG	Middle Temporal Gyrus
PDC	Partial directed coherence	Pcun	Precuneus
MC	Motor cortex	PoG	Postcentral Gyrus
dorsalA6	Dorsal Area 6	SPL	Superior Parietal Lobule
PFC	Prefrontal cortex	STG	Superior Temporal Gyrus
PMC	Premotor cortices	pSTS	Posterior Superior Temporal Sulcus
SMA	Supplementary motor area	vPM	Ventral Premotor Cortex
SVM	Support vector machines	LI	Laterality index
DWT	Discrete Wavelet Transform	FMA	Fugl-Meyer Assessment

**Table 2:** Patient demographic and clinical information

Patient No	Gender	Age at consent	Lesion side	Time since stroke (Day)	FMA Pre	FMA Post
S002-ER	M	60	R	24	28	33
S005-SOC	F	51	R	31	19	28
S007-SOC	F	57	L	26	23	33
S008-ER	M	74	L	21	20	22
S009-SOC	F	64	R	27	24	33
S012-SOC	F	57	R	31	15	23
S013-ER	F	55	L	16	32	33
S015-SOC	M	72	R	32	31	31
S016-ER	M	39	R	36	13	25
S017-SOC	F	45	R	26	26	32
S018-ER	M	54	R	38	22	25
S020-SOC	F	69	R	18	26	28
S021-ER	M	65	R	20	31	34
S022-ER	F	53	L	29	23	27
S023-SOC	M	51	L	27	32	34
S024-SOC	M	73	L	38	24	29
S025-SOC	M	60	L	36	23	23
S026-ER	M	67	L	14	23	25
S028-SOC	F	49	L	28	23	23
S029-ER	F	67	L	38	21	25
S030-SOC	M	58	R	39	27	27
S031-SOC	M	63	L	31	21	26

## Data Availability

The data that support the findings of this study are available from the corresponding author upon reasonable request.
